# Wideband RCS reduction due to plasma generated by radioactive nuclei for cylindrical object

**DOI:** 10.1038/s41598-022-16336-7

**Published:** 2022-07-14

**Authors:** M. Ramezani, R. Razavi

**Affiliations:** Department of Physics, Faculty of Science, Imam Hossein Comperhensive University, Tehran, Iran

**Keywords:** Engineering, Physics

## Abstract

Radar cross section reduction has been one of the most important research topics in recent years. Plasma-based stealth is a method of reducing the radar cross section, which dampens the electromagnetic waves and reduces the amount of return waves. In this paper, a coating of the radioactive nucleus $^{241}{\mathrm{Am}}$ on the surface of the cylinder with a radius of 10 cm is considered and the range of the emitted alpha particles and the electron density generated in the air are obtained using the Geant4 code under standard temperature and pressure conditions. By finite element method solution, the radar cross section of the conductive cylindrical object has been simulated and extracted in the presence and absence of plasma created by alpha-particles. The obtained results show a **reduction of 5–8 dB **$\mathrm{m}^2$ in the radar cross section in the frequency range of 2–12 GHz for specific activity source of 1 Ci/$\mathrm{cm}^2$.

## Introduction

Radar cross section is the ratio of the power density reflected from the target to the density of the power transmitted. In other words, if the distance between the radar and the target is R, the intensity of the electric field reaching the target surface $E_{i}$ and the intensity of the electric field received at the receiver $E_{s}$, the radar cross section is equal to^1–8^,

1 $$\begin{aligned} \sigma =lim_{R\rightarrow \infty }4\pi R^2 | E_{s}/E_{i}|^2 \end{aligned}$$

Radar cross section is either bistatic or monostatic. Bistatic refers to the case when the transmitter and the receiver are different locations, whereas monostatic cross section is for the case when the transmitter and the receiver are at the same location^9^. Various techniques to achieve radar cross-section reduction are considered by researchers, such as: shaping, radar absorbers and plasma-based stealth technology^10–12^. The shaping and radar absorbers may not be effective, in wide range of radar frequencies^13,14^. Plasma can be used as a good absorber of electromagnetic waves over a wide range of frequencies^15–17^. A plasma absorber has several advantages, exhibiting high attenuation over a large bandwidth and the weight of the plasma is negligible. Plasma can be generated in a variety of ways, such as: electrical discharge, laser, electron gun, energetic X-ray photons, ion beams and radioactive nuclei^18–24^. Plasma generated by electrical discharge, laser and electron gun requires a power supply which may be bulky while the plasma generated by radioactive nuclei does not require a power supply. The EM wave interaction with plasma mainly depends on the physical characteristics and associated plasma parameters and the plasma density^25^. Plasma is electrically conductive over wide range of frequencies. The use of plasma to control the reflected EM wave and, hence, the RCS is possible at higher frequencies, where the conductivity of the plasma results in greater interactions of plasma and the incident EM wave. The wave is absorbed within the plasma and thus contributes toward RCS reduction^25^.

The absorption of EM wave in unmagnetized plasmas was first proposed by Vidmar. The collisional unmagnetized plasma has a complex permittivity and acts as a lossy material due to energy transfer from electrons to neutrals via collisions. Vidmar showed that a tenuous, high collisional and low density plasma can be effectively used as a broad-band absorber from VHF to X-band^26,27^. Tang et al. found that high density and high collision plasma can increase microwave absorption^28^. Liu et al. considering the three common radioactive nuclei of alpha-decay: $^{210}{\mathrm{Po}}$, $^{238}{\mathrm{Pu}}$ and $^{241}{\mathrm{Am}}$ and chose the energy of alpha-particles ejected as 5.45 MeV, which possessed no obvious differences of energy loss in air with the three common radioactive nuclei^20^. $^{241}{\mathrm{Am}}$ is the most common isotope of americium, it is radioactive, with a half-life of 432.2 years. Americium-241 has been produced in small quantities in nuclear reactors and decays mainly via alpha decay, with a weak gamma ray byproduct. The alpha decay energies are 5.486 MeV for 85% of the time, 5.443 MeV for 13% of the time, and 5.388 MeV for the remaining 2%^29^.The FDTD method was used to calculate the radar cross section of the spherical body and reduction of 25.5 dB in radar cross section caused by radioactive nuclei of alpha-decay coating with activity of 10Ci/$\mathrm{cm}^2$ was abserved^19^. Dehghan et al. simulated the RCS reduction of the flat plate with plasma coating caused by alpha particles, which the obtained result shows decrease of 7–11 dB $\mathrm{m}^2$ compared without plasma coating^1^. In reference^1^, the simple geometry of the flat plate is considered and the number of plasma layers and mesh are limited, while in this paper a complex geometry of the number of plasma layers and optimal mesh are used.

In this paper, the effect of plasma generated by radioactive nuclei on reducing the radar cross section of a conductive cylinder with a radius of 10 cm has been investigated.

## Summary of the theory and discussion

**Figure 1:**
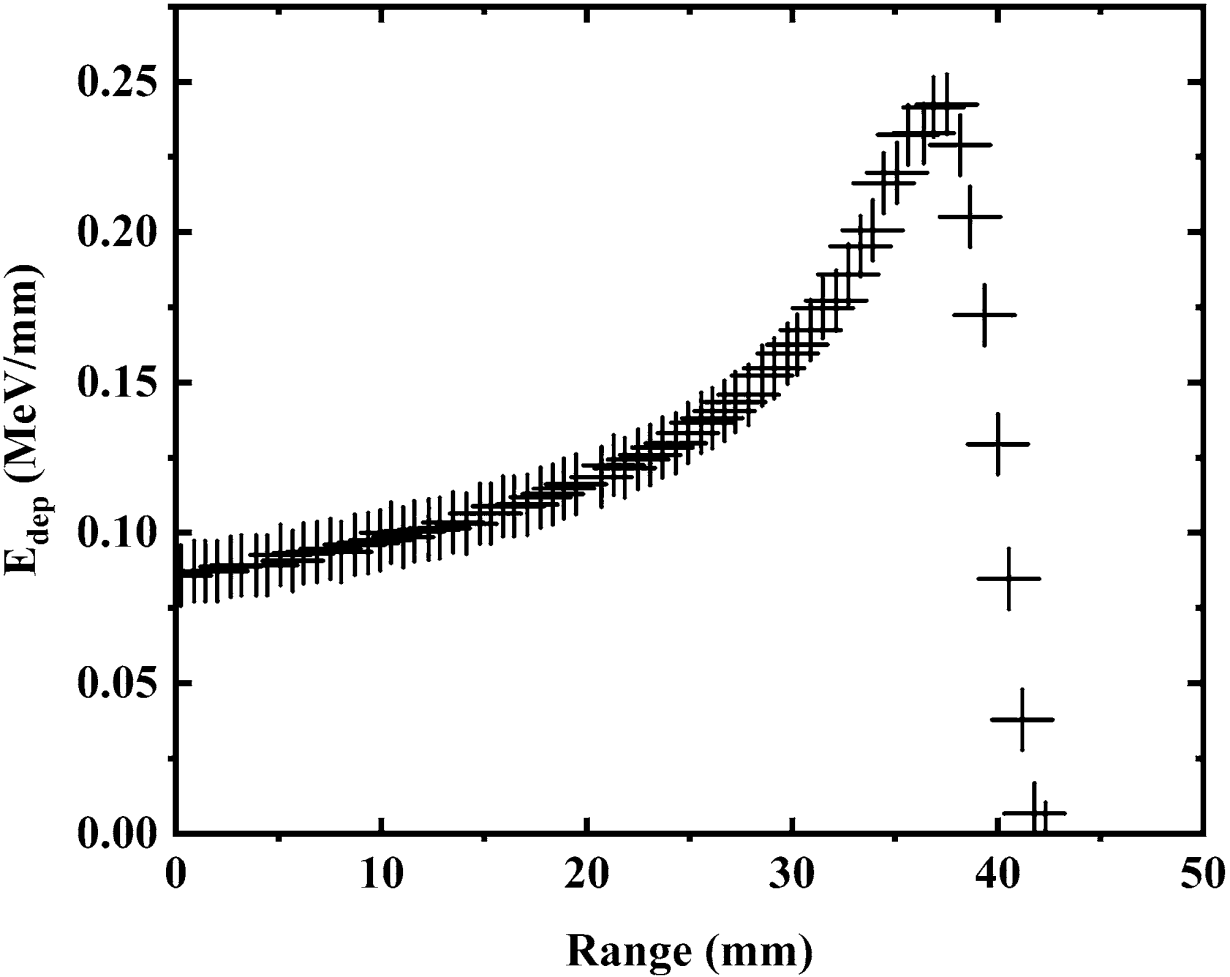
Range of alpha particles in air obtained using Geant 4 toolkit.

The radioactive nuclei $^{241}{\mathrm{Am}}$ considered to emit alpha particles with an energy of 5.45 MeV and these particles are able to ionize the surrounding air and produce a high density of electrons in layer adjacent to the surface. The deposition energies of the alpha particles emitted from the radioactive source $^{241}{\mathrm{Am}}$ in the air are obtained using the Geant4 code and shown in Fig. 1.

Geant4 is a toolkit for simulating the passage of particles through matter and capable of handling all physics processes including electromagnetic, hadronic and nucleus-nucleus intractions which are indispensable to calculate three-dimensional dose distributions and deposition energies in air and ion therapy^30^. The simulation in Geant4 code is done by determining the three main classes DetectorConstruction, PrimaryGeneratorAction and Physicslist. In class DetectorConstruction, defines simulation components such as geometry and material which in this work, the geometry of the cylinder and the material of the air are considered. In class PrimaryGeneratorAction, the quality and quantity of radiation entering the geometry of the problem must be determined which in this work, the alpha particle with an energy of 5.45 MeV is considered and in class Physicslist, event physics is determined, in this simulation, physicslistEmStandard is used. Geant4 electromagnetic physics manages the electromagnetic interactions of leptons, photons, hadrons and ions^30^.

The composition of air (G4-AIR) used in the calculations is extracted from the NIST manger library, the Geant4 code, which includes: nitrogen (0.755268), oxygen (0.231781), argon (0.012827) and carbon(0.000124).

**Figure 2:**
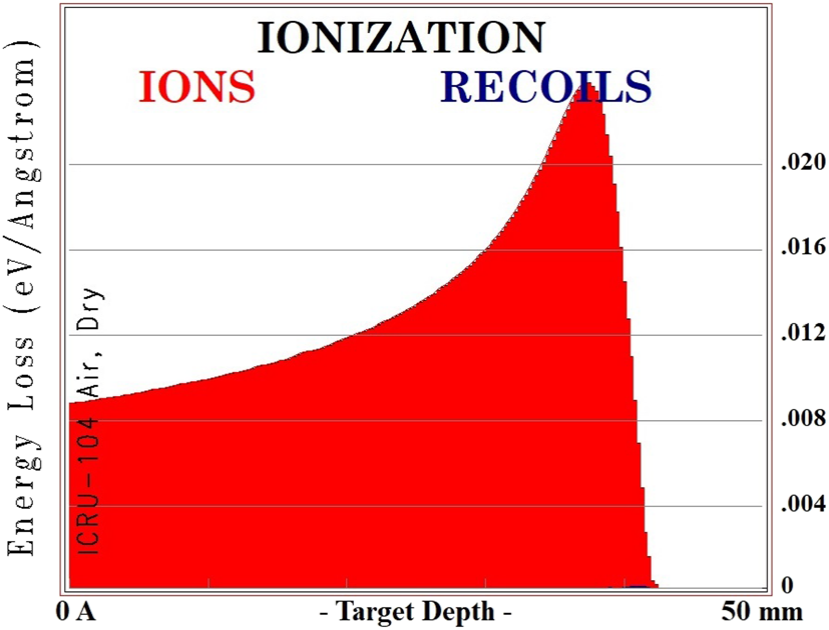
Range of alpha particles in air obtained using SRIM software.

To validate the obtained results from Geant4, the amount of deposition energy in terms of distance from the surface caused by an alpha particle was calculated using the SRIM software package (Fig. 2). The SRIM Monte Carlo simulation code is widely used to compute a number of parameters relevant to ion beam implantation and ion beam processing of materials^31^.

According to Fig. 2, the deposition energy of alpha particles is shown and the range of alpha particles is about 40 mm in the air. Dividing the amount of deposition energy by the production of each ion pair (36.08 eV), the number of electron-ion pairs produced are extracted per passage of one alpha particle in the air. The electron density of the cylinder surroundings is expressed as^32,33^,

2 $$\begin{aligned} n_{e}= \sqrt{\frac{q}{c_r}}, \end{aligned}$$

the recombination coefficient ${c_r}$ for air in the standard temperature and pressure (STP) condition is ${c}_{r}=5.75\times 10^{-9}\mathrm{cm}^{-3}/s. e$^33^.

Electron production rate for an alpha emitter isotope obtained on the lateral surface of cylinder is

3 $$\begin{aligned} q = {5N_\alpha }/2\pi \int ^{10}_{0}\frac{n({R}^{'})2\pi h d{r}}{{R}^{'2}}, \end{aligned}$$

where ${R}^{'}$ is the distance from the conductive surface, $n({R}^{'})$ is the number of electron-ion pairs produced in terms of distance ${R}^{'}$, $h = 20\,\mathrm{cm}$ is the height of the cylinder and ${N_\alpha }$ is the number of decays in terms of $\mathrm{Bq}/\mathrm{cm}^{2}$.

By placing the amount of electron density obtained in Eq. (4), the plasma frequency is calculated in terms of rad/s^34–38^:

4 $$\begin{aligned} \omega _{pe}= \sqrt{\frac{n_e e^2}{m_e\epsilon _0}}, \end{aligned}$$

where ${n_e}$ is the electron density, the electron charge e is equal to ${1.6\times 10}^{-19}{C}$, $m_e$ is the electron mass equal to ${ 9.1}\times {10}^{-31} {\mathrm{kg}}$ and ${\epsilon _0}$ is electrical permeability of vacuum space.

**Figure 3:**
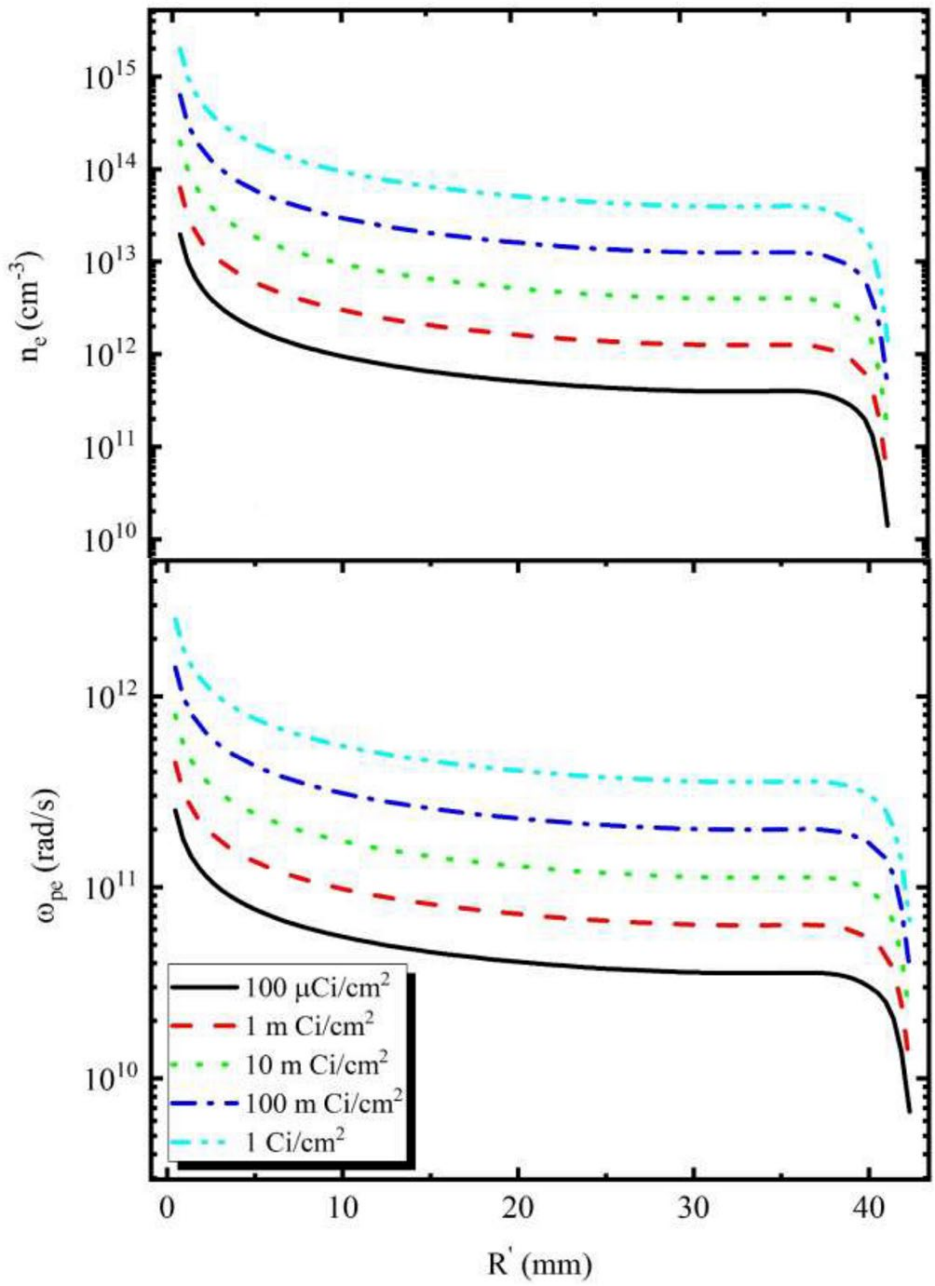
Electron density and plasma frequency of surroundings around the cylinder.

Electron density and plasma frequency for different source activities in different cells at the surface around the cylinder are shown in Fig. 3.

Alpha particles are short-range and move directly in the environment^29^. When they enter the environment, they lose their energy through ionization and excitation. It seems that at a distance of 5 to 40 mm due to the thermal balance of alpha particles with the environment, a constant ionization rate is observed, and this phenomenon causes the electron density graph to be constant in terms of range.

According to Fig. 3, for all activities used in the calculations, the electron densities have maximum values near the surface of the cylinder and the values reach approximately constant at the distance of about 5 to 40 mm from the surface of the cylinder. Finaly, the electron density decreases sharply at distances greater than 40 mm and the plasma frequency for the maximum activity (1 Ci/$\mathrm{cm}^2$) at very close distances to the surface of the geometry is $2\times 10^{12}$ and the distance between 5 and 40 mm from the surface, the value reaches approximately constant of $9\times 10^{11}$ and at distances greater than 40 mm, the plasma frequency decreases sharply.

The collision frequency is^39^.

5 $$\begin{aligned} \upsilon _{c}=8.3\times 10^5\pi a_{air}^2 \sqrt{{T}{n_0}}, \end{aligned}$$

where $$ a_{air}$$ is the radius of the air molecule $$ (4.845\times 10^{-8} \mathrm{cm}$$)^40^, T is the temperature (273 K) and $$ n_{0}$$ is the neutral gas density in ($$1/\mathrm{cm}^{3}$$). Loschmidt^40^ obtained the diameter of an air molecule using chemical methods and determining a specific volume (nitrogen 77% and oxygen 23%) as follows,

6 $$\begin{aligned} S=8\epsilon L, \end{aligned}$$

Here S is the diameter of the gas molecule, $\epsilon$ is the condensation coefficient of gas and L is the mean free path.

Collision frequency includes four types of collisions: the electrons and molecules, the electrons and ions, the ions and molecules, and the electrons and electrons^19^. We replace the electron density of the plasma environment with the density of neutral gas molecular density, therefore the least radar cross section reduction are extracted in our results and the collision frequency is expressed as follows,

7 $$\begin{aligned} \upsilon _{c}=8.3\times 10^5\pi a_{air}^2 \sqrt{{T}{n_e}}, \end{aligned}$$

**Figure 4:**
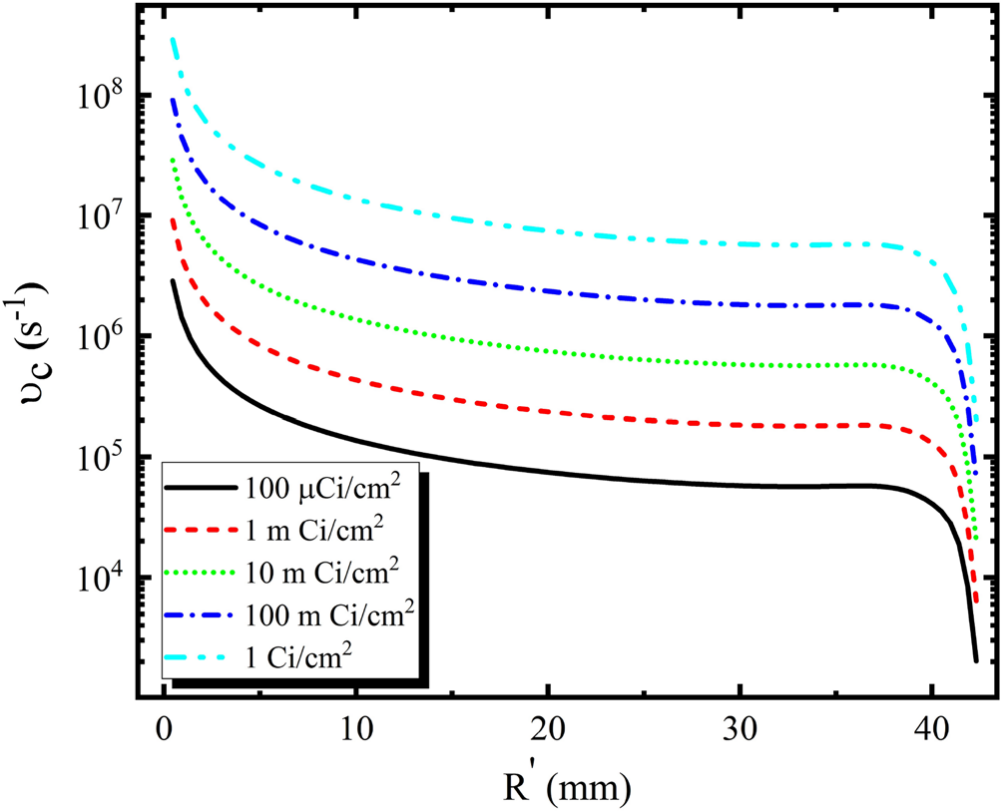
Collision frequency of surroundings around the cylinder.

The frequency of plasma collisions is determined and shown in Fig. 4.

According to Fig. 4, the collision frequency in different activities, such as electron density and plasma frequency have a similar trend.

Plasma frequency and collision frequency are important parameters that control plasma performance to reduce radar cross section (RCS). The complex dielectric permittivity of the plasma medium is given by^25^,

8 $$\begin{aligned} \epsilon _{r}=1-\frac{\omega _{p}^2}{\omega (\omega -i\upsilon _{c})}=1-\frac{\omega _{p}^2}{\omega ^2+\upsilon _{c}^2}-\frac{i\omega _{p}^2\upsilon _{c}}{\omega (\omega ^2+\upsilon _{c}^2)}, \end{aligned}$$

**Figure 5:**
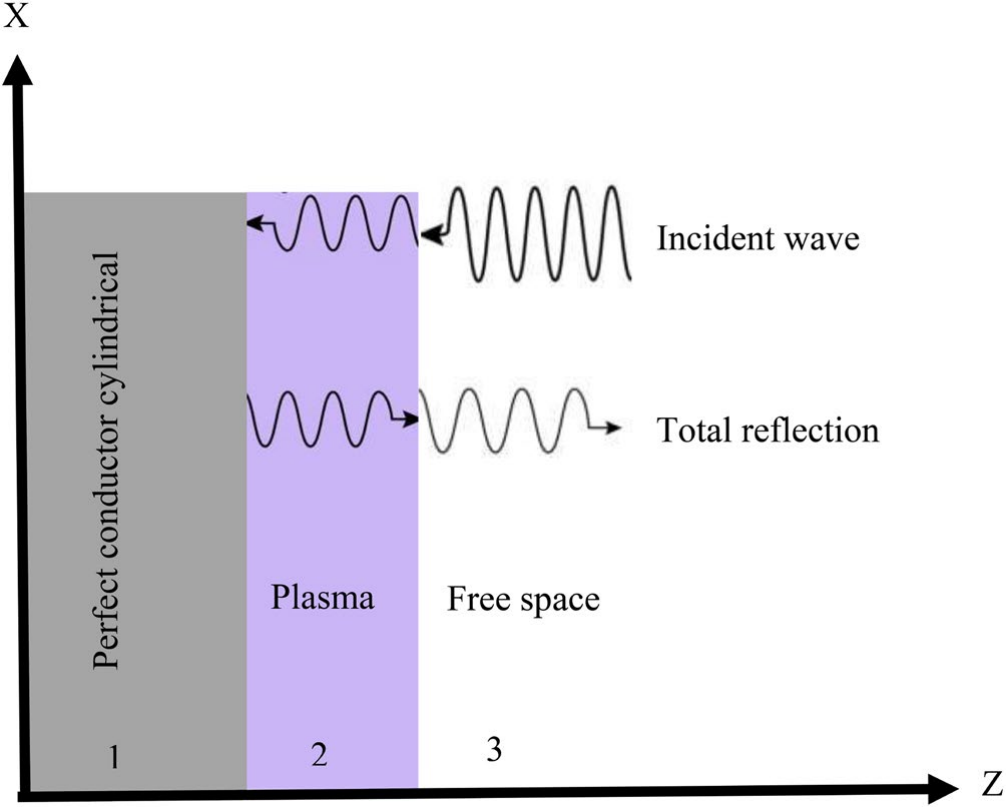
Schematic diagram of microwave reflection in the plasma.

Here $\omega _{p}$ is the plasma frequency, $\omega$ is the incident wave frequency, and $\upsilon _{c}$ is the collision frequency. The reflection of bounded plasma to determine the influences of plasma parameters on microwave transmission has been shown in Fig. 5.

**Figure 6:**
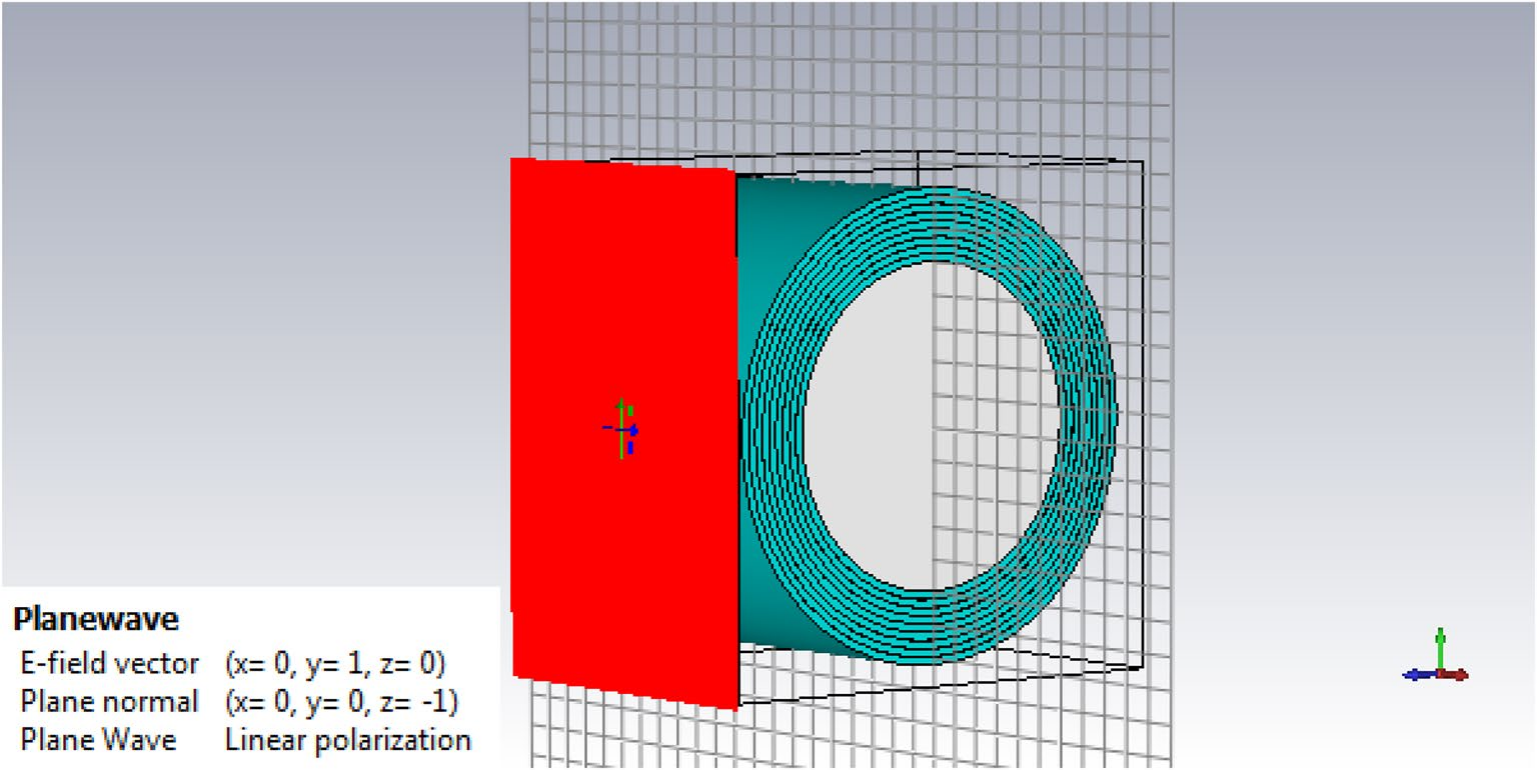
Simulation of plasma layers of surroundings around the cylinder.

In the plasma layers simulation, after defining the geometry, the Drude plug-in of the CST software, which is designed to describe cold plasma, has been used. CST is a powerful software for simulating electromagnetic fields in a three-dimensional structure. CST software includes seven modules that have the ability to simulate in the fields of electrostatic, magnetostatic, low frequency and etc^41^. One of the most widely used modules of this software is the CST Microwave Studio module. The geometry of the nine layers are modeled and the thickness of each layers are five millimeters. According to the results obtained for plasma frequency and collision frequency, the mean values of plasma frequency and collision frequency are determined for each layer of five millimeters. We place the mean values of the plasma frequency and the collision frequency in Drude module and the plasma layers are simulated. Figure 6, shows the simulated plasma layers for the environment around a conductive cylindrical body and the vertical angle of the input wave.

In this simulation, the problem is analyzed using the finite element solution method in bistatic mode.

The cylinder, because of its simplicity and the fact that its solution is represented in terms of well known and tabulated functions (such as Bessel and Hankel functions), is probably one of the geometries most widely used to represent practical scatterers. For the conducting cylinder,the three-dimensional RCS as^41,42^,

9 $$\begin{aligned} \sigma _{3-D}=\frac{4h^2}{\pi }\left|\sum \limits _{n=-\infty }^{+\infty }\frac{J_n(\beta r)}{H_n^{(2)}(\beta r)}e^{in\phi }\right|^2, \end{aligned}$$

where h is the height of the cylinder, r is the radius of the cylinder, $\beta$ is constant phase.

$H_n^{(2)}(\beta r)$ is Hankel function and defined as follows,

10 $$\begin{aligned} H_n^{(2)}(\beta r)=J_n(\beta r)-iY_n(\beta r), \end{aligned}$$

$Y_n(\beta r)$ is Neumann function and defined as follows,

11 $$\begin{aligned} Y_n(\beta r)=\frac{J_n(\beta r) cos(n\pi )-J_n(\beta r)}{sin(n\pi )}, \end{aligned}$$

$J_n(\beta r)$ is Bassel function and given by,

12 $$\begin{aligned} J_n(\beta r)=\sum \limits _{m=0}^{+\infty }\frac{(-1)^m((\beta r)/2)^{2m+n}}{m!(m+n)!}, \end{aligned}$$

**Figure 7:**
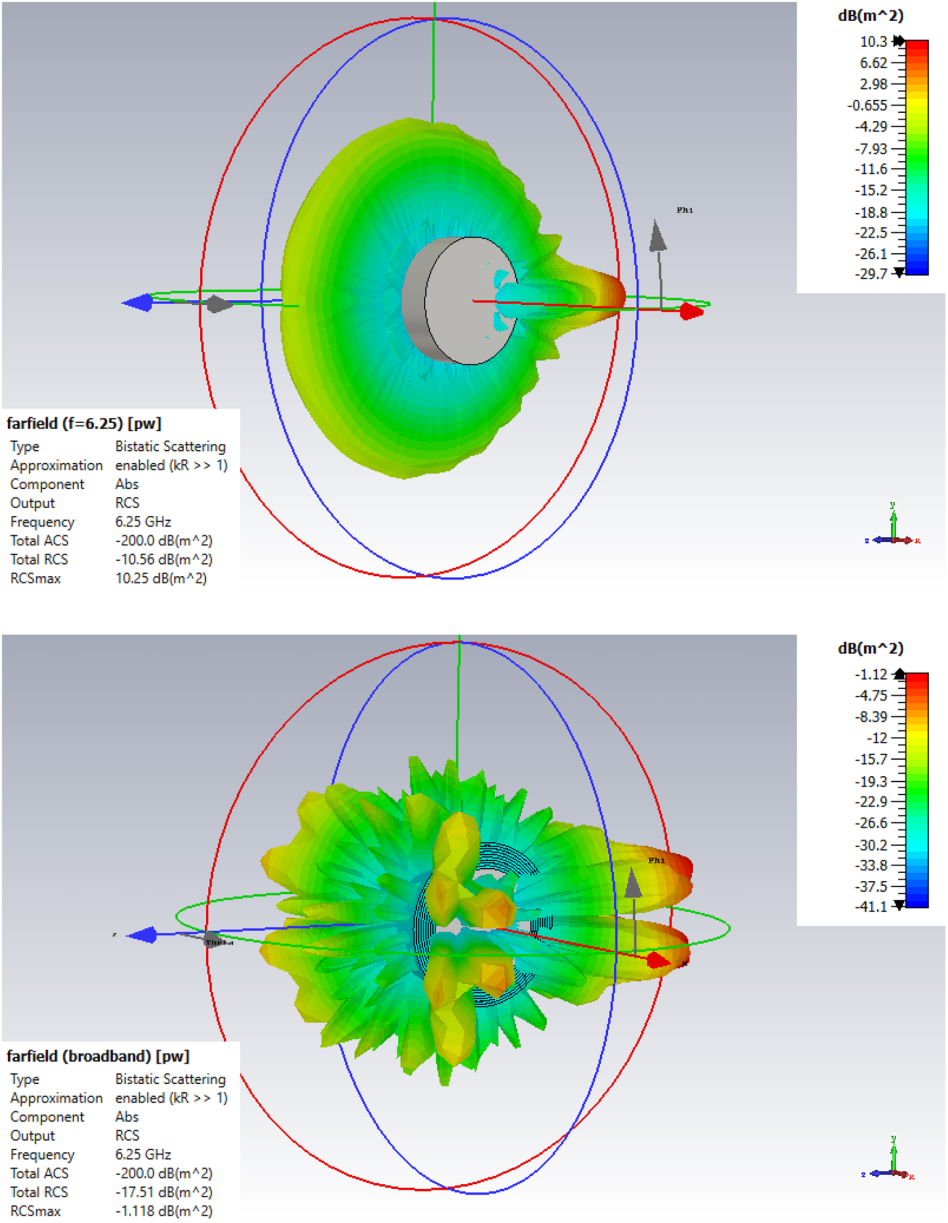
The far-field scattering 3D pattern for cylinder without radioactive nuclei (up) and with radioactive nuclei (down).

In Fig. 7, the far-field scattering 3D pattern for cylinder without and with radioactive nuclei is displayed in CST software.

**Figure 8:**
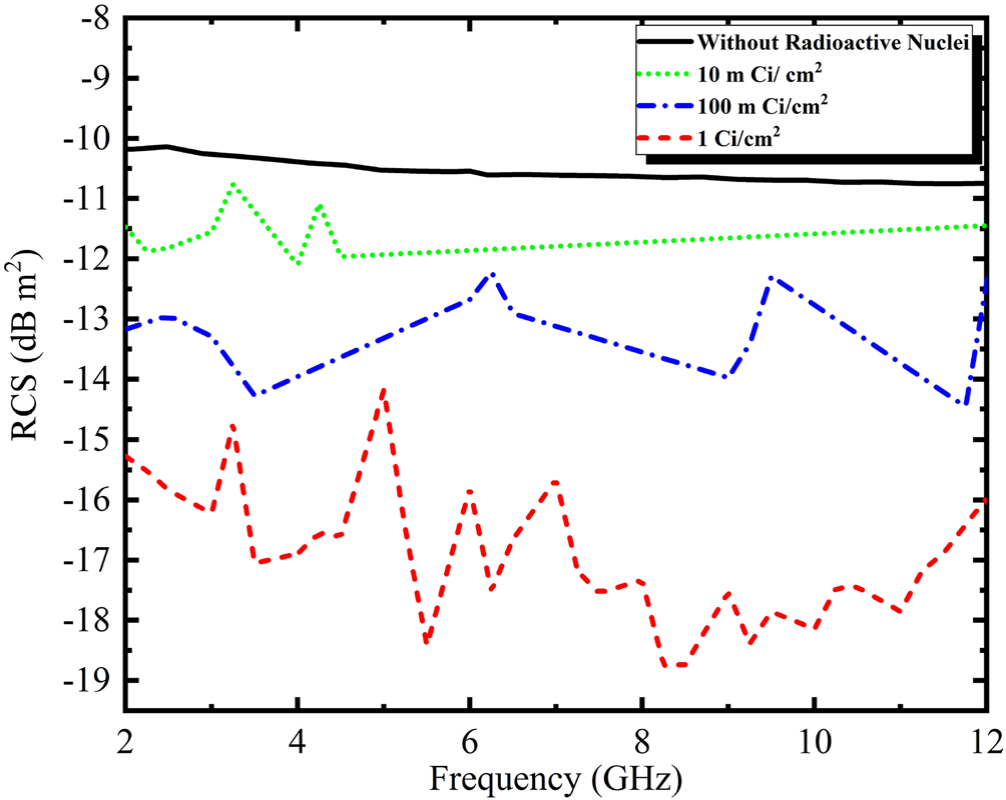
RCS results obtained from simulation of a conductive cylinder with a radius of 10 cm with coating of radioactive nuclei of amersium at different activities.

According to Fig. 7, the value of the total radar cross section in three dimensions at a frequency of 6.25 GHz without covering the radioactive nuclei and with coverage of radioactive nuclei at activity 1 Ci/$\mathrm{cm}^2$ has been extracted $-10.56$ and $-17.51$ dB $\mathrm{m}^2$ respectively, that these values correspond to Fig. 8.

Finally, the radar cross section of a conductive cylinder in the frequency range of 2 to 12 GHz with different activities of 10 mCi/$\mathrm{cm}^2$, 100 mCi/$\mathrm{cm}^2$ and 1 Ci/$\mathrm{cm}^2$ are calculated and compared without radioactive nuclei (Fig. 8).

According to the obtained results in Fig. 8, the RCS values in the frequency range of 2–12 GHz for a cylindrical body without covering the radioactive nuclei are extracted at about $-10$ dB $\mathrm{m}^2$. The RCS values are in approximate order $-11$ dB $\mathrm{m}^2$, $-13 dB$ $\mathrm{m}^2$ and $-18$ dB $\mathrm{m}^2$ for 10 mCi/$\mathrm{cm}^2$, 100 mCi/$\mathrm{cm}^2$ and 1 Ci/$\mathrm{cm}^2$ activities, respectively. The reduction of 8 dB $\mathrm{m}^2$ is observed for 1 Ci/$\mathrm{cm}^2$ activity in the frequency range of 2–12 GHz.

## Conclusions

In this paper, the effect of plasma due to alpha particles on radar cross section reduction in a cylinder has been studied and simulated. Alpha particles with a range of 41 mm cause ionization of the air around the cylindrical body. The maximum amount of electron density obtained from alpha particles in activities 10 mCi/$\mathrm{cm}^2$, 100 mCi/$\mathrm{cm}^2$ and 1 Ci/$\mathrm{cm}^2$ has been extracted from order $10^{14}$, $10^{14}$ and $10^{15}$, respectively. The maximum amount of plasma frequency and collision frequency values in activity 1 Ci/$\mathrm{cm}^2$ has been obtained from order $10^{12}$ rad/s and $10^{8}$ $s^{-1}$. The RCS obtained result in the frequency range of 2–12 GHz for the vertically flat wave on the surface of the cylindrical body with coverage of radioactive nuclei in activities 10 mCi/$\mathrm{cm}^2$, 100 mCi/$\mathrm{cm}^2$ and 1 Ci/$\mathrm{cm}^2$ show a reduction of obout 1 dB $\mathrm{m}^2$, 3 dB $\mathrm{m}^2$ and 8 dB $\mathrm{m}^2$ compared to cylinderical body without radioactive, respectively. Also, the results show the radar cross section over a wide range of frequencies, is reduced by increasing the activity.

## Data Availability

The data that support the finding of this study are available from the corresponding author upon reasonable request.
